# Longitudinal patterns of visual impairment and trajectories of cognitive function and depressive symptoms in older adults: the moderating role of social vulnerability

**DOI:** 10.3389/fpubh.2026.1899072

**Published:** 2026-07-29

**Authors:** Xia Wu, Li Feng, Li Peng, Heng Li, Ting-fan Wang

**Affiliations:** 1 Department of Otorhinolaryngology-Head and Neck Surgery, West China Hospital of Sichuan University-Ziyang Hospital, Ziyang Central Hospital, Ziyang, Sichuan, China; 2 Department of Pediatric Surgery, Affiliated Hospital of Southwest Medical University, Lu Zhou, Sichuan, China

**Keywords:** cognitive decline, depressive symptoms, loneliness, social isolation, visual impairment

## Abstract

**Background:**

Most studies treat visual impairment (VI) as a static, binary exposure. Less is known about whether distinct longitudinal VI patterns are differentially associated with later cognitive and affective trajectories, and whether social vulnerability modifies these associations.

**Objective:**

To examine whether longitudinal VI patterns classified by directionality and chronicity are differentially associated with trajectories of cognitive function and depressive symptoms, and whether social vulnerability modifies these associations.

**Methods:**

We analyzed data from 15,393 older adults in the Health and Retirement Study. VI was classified into four patterns (Never, Worsening, Improving, Persistent) based on self-reported visual status in 2006 and 2008. Cognitive function and depressive symptoms were assessed biennially from 2008 to 2020 and 2022, respectively. Linear mixed-effects models estimated trajectories adjusting for demographic, socioeconomic, and health covariates. Three-way interactions tested effect modification by loneliness, living alone, and partner status. Benjamini-Hochberg false discovery rate correction was applied to the three-way interaction analyses.

**Results:**

All VI groups had lower baseline cognitive scores and higher depressive symptoms than Never VI. The Improving VI group showed the steepest cognitive decline (*β* = −0.051/wave, 95% CI: −0.100 to −0.002, *p* = 0.040). Persistent VI had the highest baseline depressive symptoms but showed a relative decline over follow-up (*β* = −0.035/wave, *p* = 0.002). After correction for multiple testing, partner status attenuated cognitive decline in the Improving VI group, and partner status, living arrangement, and loneliness modified selected depressive symptom trajectories.

**Conclusion:**

Longitudinal VI patterns carry distinct prognostic significance for cognitive and affective trajectories. Improving VI was associated with modestly faster subsequent cognitive decline (cumulative difference approximately 0.31 points over 12 years, or 1.1% of the cognitive scale), warranting continued monitoring even after apparent visual improvement. Social vulnerability modified selected VI-related trajectories, supporting the need for future research on integrated sensory and social intervention strategies.

## Introduction

Visual impairment affects approximately one in three adults aged 65 and older and is projected to increase substantially as populations age worldwide (1). Beyond its direct impact on functional independence, VI has been linked to accelerated cognitive decline (2, 3) and elevated depressive symptoms (4, 5) in older adults. These associations are hypothesized to operate through multiple pathways, including reduced sensory input to the brain, diminished social engagement, and loss of cognitively stimulating activities (6, 7).

Despite growing evidence linking VI to adverse health outcomes, two critical gaps remain. First, the majority of studies have classified VI as a binary, time-fixed exposure measured at a single assessment (2, 3). This approach treats all individuals with VI as sharing a common risk profile, ignoring the heterogeneity inherent in the natural course of visual function in later life. Vision may worsen progressively, improve following cataract surgery or corrective intervention (8), or remain chronically impaired, trajectories that imply fundamentally different clinical narratives. A patient whose vision is restored through cataract surgery does not share the same risk profile as someone with progressive, untreatable vision loss, yet binary classifications collapse these distinct experiences into a single category (8). Moreover, the possibility that apparent improvement in visual function may not uniformly translate into cognitive benefit has received limited longitudinal attention. Whether the directionality and chronicity of VI change carry distinct prognostic significance for subsequent cognitive and affective trajectories remains largely unexamined.

Second, the role of social vulnerability in modifying the relationship between VI and health decline is poorly understood. Older adults with VI are at elevated risk of social isolation and loneliness (6, 9)—factors that independently predict cognitive decline (7, 10) and depression (11, 12). In this study, social vulnerability refers to limited social resources or social connectedness that may reduce an older adult’s capacity to cope with sensory loss. We operationalized this construct using three complementary indicators: loneliness as a marker of perceived social disconnection, living arrangement as a marker of structural isolation, and partner status as a marker of close emotional and instrumental support. Social vulnerability may magnify the downstream consequences of VI by compounding reductions in environmental stimulation and emotional support (13, 14), yet virtually no studies have formally tested whether loneliness, living arrangement, or partner status modify the longitudinal health effects of VI.

To address these gaps, we used data from the Health and Retirement Study, a nationally representative longitudinal cohort of U.S. adults aged 50 and older (15), to examine associations between longitudinal VI patterns and trajectories of cognitive function and depressive symptoms over up to 14 years of follow-up. Participants were classified into four mutually exclusive VI patterns based on visual status across two consecutive waves (2006 and 2008): Never VI, Worsening VI, Improving VI, and Persistent VI. This exposure window was selected because it was the earliest period in which visual status across two consecutive waves and psychosocial data from the Leave-Behind Questionnaire were both available, allowing concurrent assessment of loneliness as a social modifier before the full outcome follow-up period (16, 17). We examined whether these patterns were differentially associated with subsequent health trajectories and whether social vulnerability, indexed by loneliness, living alone, and partner status, modified these associations.

We tested the following hypotheses:

- Persistent and Worsening VI would be associated with steeper cognitive decline and greater increases in depressive symptoms relative to Never VI.
- Social vulnerability would amplify adverse VI-related cognitive and affective trajectories.
- The role of directionality, specifically whether Improving VI and Worsening VI carried different prognostic significance, was examined as an exploratory aim.

## Methods

### Study population and data source

Data were drawn from the Health and Retirement Study, a nationally representative prospective longitudinal cohort of U.S. adults aged 50 and older (15). We used the RAND HRS Longitudinal File, a harmonized version of the HRS data. The study baseline was set at 2008, with cognitive function tracked biennially through 2020 (seven assessments) and depressive symptoms through 2022 (eight assessments). The 2006–2008 window was selected because Wave 8 marked the first HRS wave with consistent availability of the psychosocial Leave-Behind Questionnaire (16), enabling concurrent assessment of loneliness as a social modifier. This exposure window was therefore selected primarily on the basis of data availability, rather than *a priori* clinical or biological considerations.

**Figure 1:**
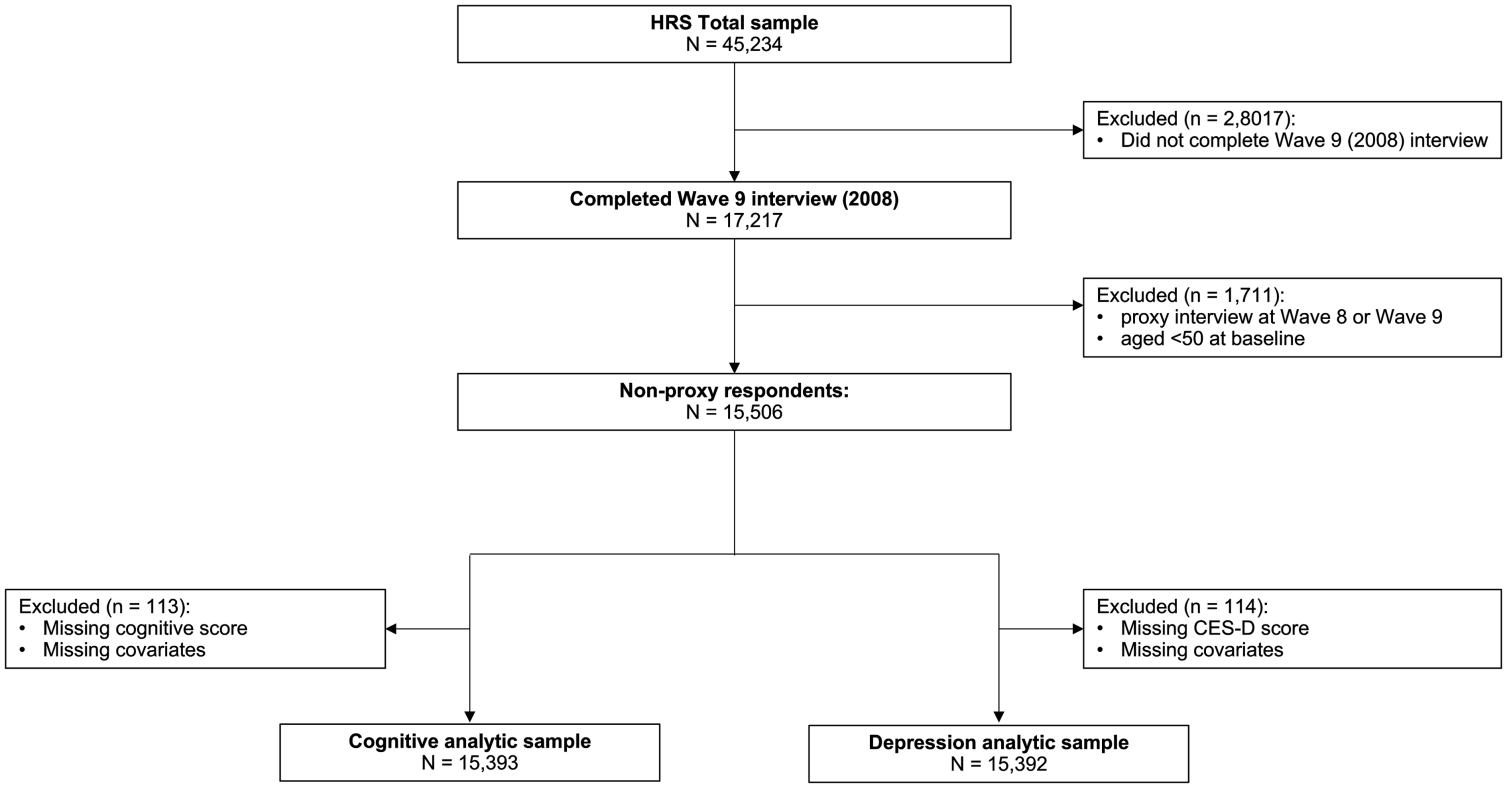
Flow diagram of study participant selection. HRS, Health and Retirement Study. Wave 8 = 2006; Wave 9 = 2008. Non-proxy respondents were defined as participants who completed the interview without a proxy respondent at both Wave 8 and Wave 9. The cognitive analytic sample required a non-missing baseline cognitive composite score at Wave 9; the depression analytic sample required a non-missing CES-D score at Wave 9. Both samples required complete data on all key covariates.

Participants were included if they completed the Wave 9 (2008) interview, were aged 50 years or older at baseline, and provided valid self-reported visual acuity data at both Wave 8 (2006) and Wave 9 (2008) to permit classification of longitudinal VI patterns. Participants who completed interviews via proxy at either wave were excluded, as proxy-reported data may not reliably capture self-perceived visual acuity or cognitive performance. Participants missing baseline outcome measures or key covariates were further excluded. Accordingly, baseline covariates were handled using complete-case analysis. Loneliness data were not required for inclusion in the main analytic sample, as loneliness was treated as an effect modifier rather than a confounder; participants with missing loneliness data were excluded only from the loneliness interaction models. The final analytic samples comprised 15,393 participants for cognitive analyses and 15,392 for depression analyses (Figure 1).

### Measures

#### Visual impairment patterns (exposure)

Self-reported distance eyesight was rated on a five-point scale (1 = poor to 5 = excellent). VI was defined as eyesight rated poor or fair (≤2). Based on VI status in 2006 and 2008, participants were categorized into four mutually exclusive longitudinal patterns: Never VI (no VI in either wave), Worsening VI (no VI in 2006, VI in 2008), Improving VI (VI in 2006, no VI in 2008), and Persistent VI (VI in both waves).

### Outcomes

Cognitive function was assessed using the 27-point HRS composite score, which combines immediate and delayed word recall (0–20 points), serial sevens subtraction (0–5 points), and backwards counting (0–2 points) (18). Depressive symptoms were measured using the eight-item Center for Epidemiologic Studies Depression scale (range: 0–8) (19), with higher scores indicating greater symptom severity.

### Social vulnerability modifiers

Three social vulnerability modifiers were assessed at baseline (2008): partner status (partnered vs. not partnered), living arrangement (living alone vs. not living alone), and loneliness. These indicators were selected to capture complementary dimensions of social vulnerability: partner status as a marker of close emotional and instrumental support, living arrangement as a marker of structural social isolation, and loneliness as a marker of perceived social disconnection. Loneliness was measured using the three-item UCLA Loneliness Scale from the HRS Leave-Behind Questionnaire (17). The RAND HRS stores this measure as the mean of the three items (range: 1–3), which is equivalent to a sum score range of 3–9. Owing to the split-sample design of the Leave-Behind Questionnaire (16), 2008 values were supplemented with 2006 data where missing. A mean score of 2 or greater was defined as high loneliness; given the three-item scale, this threshold is equivalent to a sum score of 6 or greater (on a 3–9 range).

### Covariates

Baseline covariates included age, sex, race and ethnicity (Non-Hispanic White, Non-Hispanic Black, Hispanic, Other), educational attainment (less than high school, high school graduate, some college, college or above), log-transformed household wealth (20) (using the signed log transformation, sign(x) × log(|x| + 1), to accommodate negative net worth values), self-reported hearing impairment (defined as poor or fair hearing, ≤2 on a five-point scale) (21, 22), diabetes, and current smoking status. Time-varying comorbidity burden, defined as the sum of eight chronic conditions (hypertension, diabetes, cancer, lung disease, heart disease, stroke, arthritis, and psychiatric conditions) and updated at each wave, was included in all models. ADL and IADL limitations were treated as potential mediators of the VI–health relationship and were adjusted for only in sensitivity analyses. All continuous predictors were standardized (z-scores) prior to model estimation.

### Statistical analysis

Linear mixed-effects models with participant-specific random intercepts were used to estimate trajectories of cognitive function and depressive symptoms over time (23, 24). We initially attempted to fit models with participant-specific random slopes for time using both correlated and uncorrelated random-effects structures. Although these models converged, they consistently yielded singular fits, with the random slope variance estimated at or near the boundary (effectively zero). We therefore retained participant-specific random intercepts only as the most parsimonious and empirically supported specification. This specification may yield underestimated standard errors for time-varying effects if residual between-subject heterogeneity in rates of change remains unmodeled. Time was coded as a continuous variable (0 = 2008 baseline), incremented by one unit per biennial wave. Under the missing-at-random assumption, linear mixed-effects models use all available observations (23).

Two complementary model sets were fitted. Model A examined the effect of binary baseline VI status (present vs. absent in 2008) on subsequent health trajectories, without adjustment for baseline outcome score, to avoid over-controlling for pre-existing differences inherent in the baseline VI classification (25). Model B used Never VI as the reference group to compare trajectories across the four longitudinal VI patterns. Unlike Model A, Model B additionally adjusted for baseline outcome score to account for pre-existing between-group differences in cognitive function and depressive symptoms.

To examine social vulnerability as an effect modifier, three-way interaction terms (VI Pattern × Time × Social Modifier) were introduced into separate models for each modifier. Loneliness data were available for 12,588 participants (81.8% of the cognitive analytic sample), reflecting the split-sample Leave-Behind Questionnaire design (16); loneliness interaction models were therefore restricted to this subsample. Because this missingness was largely design-based rather than arising solely from participant nonresponse, loneliness was analyzed in a restricted subsample rather than imputed. Participants with and without loneliness data did not differ substantially on key baseline characteristics, including mean age, sex distribution, and baseline cognitive score. To account for multiple testing across the three social vulnerability modifiers, Benjamini-Hochberg false discovery rate correction was applied separately to the nine three-way interaction terms for cognitive function (three VI patterns × three modifiers) and the nine corresponding three-way interaction terms for depressive symptoms.

### Sensitivity analyses

Two sensitivity analyses were conducted. First, models were additionally adjusted for time-varying ADL and IADL limitations, to examine the extent to which physical functional decline accounted for the observed associations. Second, inverse probability of censoring weighting was applied to address potential informative dropout (26, 27). IPCW weights were estimated via logistic regression models predicting observation at each wave, conditional on age, sex, race and ethnicity, education, log-transformed household wealth, hearing impairment, diabetes, smoking, VI pattern, and baseline outcome score, with weights truncated at the 99th percentile (26).

Attrition analyses compared baseline characteristics between completers and non-completers to characterize patterns of selective attrition (27). Because non-completers were older and had lower baseline cognitive scores, higher depressive symptom scores, lower household wealth, and a higher prevalence of VI, these analyses suggested selective dropout. Although the missing-at-random assumption cannot be formally verified, the inclusion of observed predictors of missingness in the mixed-effects and IPCW models supports its plausibility. All statistical tests were two-sided with a significance threshold of *p* < 0.05. Analyses were conducted in R (version 4.5.2) using the lme4 (23) and lmerTest (24) packages.

## Results

### Sample characteristics

The cognitive analytic sample comprised 15,393 participants: 12,602 (81.9%) Never VI, 1,042 (6.8%) Worsening VI, 881 (5.7%) Improving VI, and 868 (5.6%) Persistent VI. The depression analytic sample comprised 15,392 participants with a nearly identical distribution. Compared with the Never VI group, participants with any VI pattern were older, more likely to be female, and had lower educational attainment and household wealth (all *p* < 0.001). The Persistent VI group had the highest burden of comorbidity, hearing impairment, and depressive symptoms at baseline. High loneliness was most prevalent in the Persistent VI group (43.3%) and lowest in the Never VI group (24.2%), with Worsening VI (35.8%) and Improving VI (35.8%) at intermediate levels (Table 1).

**Table 1:** Baseline characteristics of the study sample by longitudinal visual impairment pattern.

Characteristic	Total (*N* = 15,393)	Never VI (*n* = 12,602)	Worsening VI (*n* = 1,042)	Improving VI (*n* = 881)	Persistent VI (*n* = 868)	*p*-value
Age, years, mean (SD)	69.28 (10.02)	68.80 (9.77)	71.12 (10.59)	70.88 (10.52)	72.30 (11.36)	<0.001
Log household wealth, mean (SD)	10.81 (4.95)	11.21 (4.56)	9.35 (5.92)	9.29 (5.96)	8.36 (6.42)	<0.001
Loneliness score, mean (SD)^a^	1.48 (0.54)	1.45 (0.52)	1.61 (0.60)	1.63 (0.60)	1.70 (0.62)	<0.001
Baseline cognitive score, mean (SD)	15.06 (4.57)	15.50 (4.42)	13.19 (4.72)	13.43 (4.72)	12.54 (4.63)	<0.001
Baseline CES-D score, mean (SD)	1.44 (1.97)	1.24 (1.80)	2.20 (2.38)	2.11 (2.27)	2.77 (2.42)	<0.001
Female, *n* (%)	9,243 (60.0)	7,401 (58.7)	675 (64.8)	574 (65.2)	593 (68.3)	<0.001
*Race/ethnicity, n (%)*						<0.001
Non-Hispanic White	11,548 (75.0)	9,816 (77.9)	660 (63.3)	563 (63.9)	509 (58.6)	
Non-Hispanic Black	2,119 (13.8)	1,591 (12.6)	182 (17.5)	178 (20.2)	168 (19.4)	
Hispanic	1,386 (9.0)	910 (7.2)	182 (17.5)	124 (14.1)	170 (19.6)	
Other	340 (2.2)	285 (2.3)	18 (1.7)	16 (1.8)	21 (2.4)	
*Education, n (%)*						<0.001
Less than high school	3,809 (24.7)	2,631 (20.9)	425 (40.8)	345 (39.2)	408 (47.0)	
High school graduate	4,771 (31.0)	3,944 (31.3)	298 (28.6)	279 (31.7)	250 (28.8)	
Some college	2,747 (17.8)	2,366 (18.8)	163 (15.6)	117 (13.3)	101 (11.6)	
College or above	4,066 (26.4)	3,661 (29.1)	156 (15.0)	140 (15.9)	109 (12.6)	
Hearing impairment, *n* (%)	3,213 (20.9)	2,160 (17.1)	392 (37.6)	266 (30.2)	395 (45.5)	<0.001
Diabetes, *n* (%)	3,160 (20.5)	2,340 (18.6)	294 (28.2)	260 (29.5)	266 (30.6)	<0.001
Current smoking, *n* (%)	1,989 (12.9)	1,563 (12.4)	153 (14.7)	144 (16.3)	129 (14.9)	<0.001
Partnered, *n* (%)	9,771 (63.5)	8,281 (65.7)	581 (55.8)	465 (52.8)	444 (51.2)	<0.001
Living alone, *n* (%)	3,842 (25.0)	3,030 (24.0)	292 (28.0)	266 (30.2)	254 (29.3)	<0.001
High loneliness, *n* (%)^a^	3,471 (27.6)	2,607 (24.2)	302 (35.8)	261 (35.8)	301 (43.3)	<0.001

VI, visual impairment. Never VI: no VI at either wave; Worsening VI: no VI in 2006, VI in 2008; Improving VI: VI in 2006, no VI in 2008; Persistent VI: VI at both waves. VI was defined as self-reported distance eyesight rated poor or fair (≤2 on a 5-point scale from 1 = poor to 5 = excellent). Hearing impairment was defined as self-reported hearing rated poor or fair (≤2 on a 5-point scale from 1 = poor to 5 = excellent). Baseline characteristics are presented for the cognitive analytic sample (*N* = 15,393); the depression analytic sample (*N* = 15,392) had nearly identical characteristics. *p*-values are from one-way ANOVA for continuous variables and chi-square tests for categorical variables. CES-D, Center for Epidemiologic Studies Depression scale.

a Loneliness data available for *n* = 12,588 (81.8%); high loneliness defined as mean score ≥2 on the 3-item UCLA Loneliness Scale. Percentages for high loneliness are calculated among those with available data.

### Visual impairment and cognitive trajectories

In Model A, baseline VI was associated with significantly lower cognitive scores (*β* = −0.653, 95% CI: −0.825 to −0.481, *p* < 0.001). The VI × Time interaction was not significant (*β* = −0.015, 95% CI: −0.051 to 0.021, *p* = 0.421), indicating no difference in the rate of cognitive decline by baseline VI status (Table 2).

**Table 2:** Associations between baseline visual impairment status and longitudinal trajectories of cognitive function and depressive symptoms (Model A).

Parameter	Cognitive function *β* (SE) [95% CI]	*p*-value	Depressive symptoms *β* (SE) [95% CI]	*p*-value
VI status at baseline (ref: No VI)				
Visual impairment	−0.653 (0.088) [−0.825, −0.481]	<0.001	0.677 (0.040) [0.598, 0.756]	<0.001
Time (per biennial wave)	−0.292 (0.006) [−0.304, −0.279]	<0.001	−0.011 (0.003) [−0.016, −0.005]	<0.001
VI × Time interaction	−0.015 (0.019) [−0.051, 0.021]	0.421	−0.019 (0.008) [−0.035, −0.004]	0.014
Covariates				
Age (z-score)	−1.842 (0.028) [−1.896, −1.788]	<0.001	−0.027 (0.013) [−0.052, −0.003]	0.030
Female	0.679 (0.052) [0.577, 0.781]	<0.001	0.338 (0.024) [0.291, 0.384]	<0.001
NH Black (ref: NH White)	−2.282 (0.077) [−2.432, −2.132]	<0.001	0.013 (0.035) [−0.055, 0.082]	0.704
Hispanic	−1.412 (0.094) [−1.595, −1.228]	<0.001	0.381 (0.043) [0.297, 0.465]	<0.001
Other	−1.456 (0.169) [−1.787, −1.125]	<0.001	0.337 (0.077) [0.186, 0.488]	<0.001
HS graduate (ref: <HS)	1.947 (0.071) [1.807, 2.087]	<0.001	−0.320 (0.033) [−0.384, −0.256]	<0.001
Some college	2.760 (0.081) [2.601, 2.919]	<0.001	−0.376 (0.037) [−0.448, −0.303]	<0.001
College or above	3.647 (0.077) [3.496, 3.799]	<0.001	−0.512 (0.035) [−0.581, −0.443]	<0.001
Log household wealth (z-score)	0.357 (0.027) [0.304, 0.410]	<0.001	−0.193 (0.012) [−0.217, −0.168]	<0.001
Hearing impairment	−0.545 (0.065) [−0.673, −0.418]	<0.001	0.399 (0.030) [0.341, 0.457]	<0.001
Diabetes	−0.292 (0.068) [−0.424, −0.159]	<0.001	−0.120 (0.031) [−0.181, −0.060]	<0.001
Current smoking	−0.566 (0.077) [−0.718, −0.415]	<0.001	0.397 (0.035) [0.328, 0.466]	<0.001
Comorbidity burden	−0.143 (0.015) [−0.173, −0.113]	<0.001	0.279 (0.007) [0.265, 0.292]	<0.001

VI, visual impairment; SE, standard error; CI, confidence interval. Models used linear mixed-effects models with participant-specific random intercepts. Time was coded as 0 at baseline (2008), incremented by 1 per biennial wave. The VI × Time interaction term tests whether baseline VI status modifies the rate of change over time. Cognitive function was assessed using the 27-point HRS composite score (Waves 9–15, 2008–2020). Depressive symptoms were assessed using the 8-item CES-D scale (Waves 9–16, 2008–2022). All continuous predictors were standardized (z-scores). Comorbidity burden is a time-varying covariate updated at each wave.

**Figure 2:**
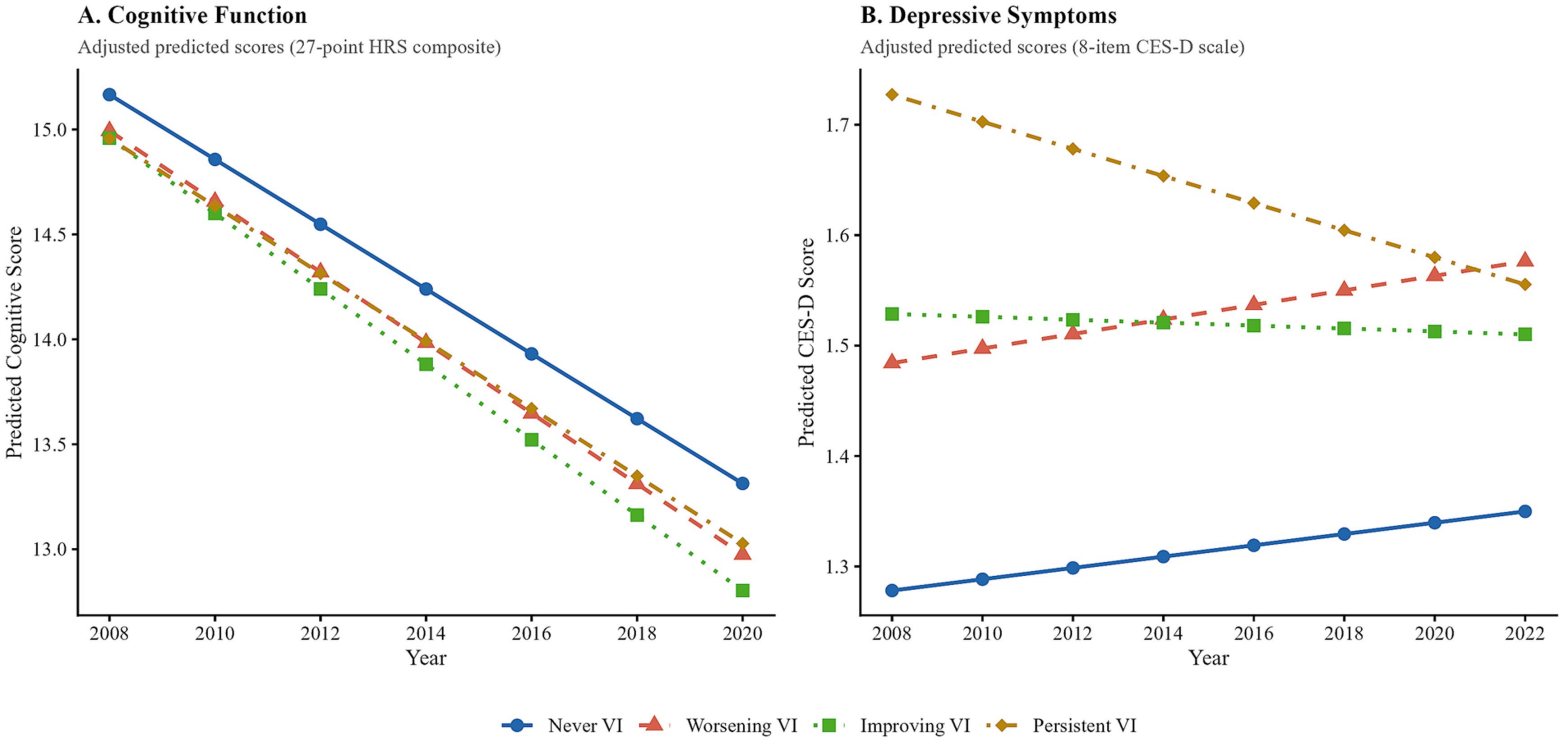
Adjusted predicted trajectories of cognitive function and depressive symptoms by longitudinal visual impairment pattern. Predicted values were derived from linear mixed-effects Model B, adjusted for age, sex, race/ethnicity, education, log household wealth, hearing impairment, diabetes, current smoking, and time-varying comorbidity burden. Predictions were generated with continuous covariates set to their sample means (z = 0 after standardization) and categorical covariates set to their reference categories, unless otherwise specified. Panel A shows predicted cognitive composite scores (2008–2020); Panel B shows predicted CES-D scores (2008–2022). VI, visual impairment; CES-D, Center for Epidemiologic Studies Depression scale.

In Model B, all VI pattern groups had significantly lower baseline cognitive scores relative to Never VI (Worsening VI: *β* = −0.173, 95% CI: −0.338 to −0.008, *p* = 0.039; Improving VI: *β* = −0.207, 95% CI: −0.383 to −0.031, *p* = 0.021; Persistent VI: *β* = −0.209, 95% CI: −0.391 to −0.027, *p* = 0.024). Only the Improving VI group showed a significantly steeper rate of cognitive decline relative to Never VI (*β* = −0.051 per wave, 95% CI: −0.100 to −0.002, *p* = 0.040). Neither Worsening VI (*β* = −0.028, 95% CI: −0.073 to 0.017, *p* = 0.226) nor Persistent VI (*β* = −0.013, 95% CI: −0.066 to 0.040, *p* = 0.630) differed significantly from Never VI in rate of change (Table 3; Figure 2).

**Table 3:** Associations between longitudinal visual impairment patterns and cognitive function trajectories: main model and sensitivity analyses.

Parameter	Main model β (SE)	*p*-value	Sensitivity 1 + ADL/IADL β (SE)	*p*-value	Sensitivity 2 IPCW-weighted *β* (SE)	*p*-value
VI pattern (ref: Never VI) — Baseline level						
Worsening VI	−0.173 (0.084)	0.039	−0.034 (0.083)	0.679	−0.180 (0.093)	0.054
Improving VI	−0.207 (0.090)	0.021	−0.089 (0.089)	0.316	−0.221 (0.101)	0.029
Persistent VI	−0.209 (0.093)	0.024	0.087 (0.092)	0.343	−0.219 (0.104)	0.034
Time (per biennial wave)	−0.309 (0.006)	<0.001	−0.292 (0.006)	<0.001	−0.342 (0.006)	<0.001
Baseline cognitive score	0.646 (0.005)	<0.001	0.627 (0.004)	<0.001	0.623 (0.005)	<0.001
VI pattern × Time interaction						
Worsening VI × Time	−0.028 (0.023)	0.226	−0.021 (0.023)	0.357	−0.027 (0.021)	0.208
Improving VI × Time	−0.051 (0.025)	0.040	−0.037 (0.024)	0.130	−0.048 (0.024)	0.046
Persistent VI × Time	−0.013 (0.027)	0.630	0.011 (0.026)	0.684	−0.015 (0.025)	0.546
Additional covariates						
ADL limitations	—	—	−0.104 (0.017)	<0.001	—	—
IADL limitations	—	—	−0.570 (0.019)	<0.001	—	—

VI, visual impairment; SE, standard error; ADL, activities of daily living; IADL, instrumental activities of daily living; IPCW, inverse probability of censoring weighting. All models include random intercepts per participant and adjust for age, sex, race/ethnicity, education, log household wealth, hearing impairment, diabetes, current smoking, and time-varying comorbidity burden. The main model additionally adjusts for baseline cognitive score. Sensitivity Analysis 1 additionally adjusts for time-varying ADL and IADL limitations. Sensitivity Analysis 2 applies IPCW to account for informative dropout, with weights truncated at the 99th percentile. All continuous predictors were standardized (*z*-scores). —, not applicable.

### Visual impairment and depressive symptom trajectories

In Model A, baseline VI was associated with significantly higher depressive symptom scores (*β* = 0.677, 95% CI: 0.598 to 0.756, *p* < 0.001). The VI × Time interaction was significant (*β* = −0.019, 95% CI: −0.035 to −0.004, *p* = 0.014), reflecting a modest relative decrease in depressive symptoms over time among those with baseline VI (Table 2).

In Model B, all VI pattern groups had significantly elevated baseline depressive symptoms relative to Never VI, with a clear severity gradient (Worsening VI: *β* = 0.206, 95% CI: 0.128 to 0.284, *p* < 0.001; Improving VI: *β* = 0.251, 95% CI: 0.167 to 0.335, *p* < 0.001; Persistent VI: *β* = 0.449, 95% CI: 0.363 to 0.535, *p* < 0.001). The Persistent VI group showed a significant negative trajectory relative to Never VI (*β* = −0.035 per wave, 95% CI: −0.057 to −0.013, *p* = 0.002), reflecting a relative decline in depressive symptoms over time. Neither Worsening VI (*β* = 0.003, 95% CI: −0.017 to 0.023, *p* = 0.762) nor Improving VI (*β* = −0.013, 95% CI: −0.033 to 0.007, *p* = 0.219) showed significant differential change (Table 4; Figure 2).

**Table 4:** Associations between longitudinal visual impairment patterns and depressive symptom trajectories: main model and sensitivity analyses.

Parameter	Main model β (SE)	*p*-value	Sensitivity 1 + ADL/IADL β (SE)	*p*-value	Sensitivity 2 IPCW-weighted β (SE)	*p*-value
VI pattern (ref: Never VI) — Baseline level						
Worsening VI	0.206 (0.040)	<0.001	0.137 (0.039)	<0.001	0.234 (0.045)	<0.001
Improving VI	0.251 (0.043)	<0.001	0.189 (0.042)	<0.001	0.264 (0.049)	<0.001
Persistent VI	0.449 (0.044)	<0.001	0.315 (0.043)	<0.001	0.492 (0.050)	<0.001
Time (per biennial wave)	0.010 (0.003)	<0.001	0.001 (0.003)	0.653	0.016 (0.003)	<0.001
Baseline CES-D score	0.577 (0.004)	<0.001	0.544 (0.004)	<0.001	0.535 (0.005)	<0.001
VI pattern × Time interaction						
Worsening VI × Time	0.003 (0.010)	0.762	−0.002 (0.010)	0.805	−0.004 (0.009)	0.636
Improving VI × Time	−0.013 (0.010)	0.219	−0.019 (0.010)	0.061	−0.010 (0.010)	0.312
Persistent VI × Time	−0.035 (0.011)	0.002	−0.051 (0.011)	<0.001	−0.033 (0.011)	0.002
Additional covariates						
ADL limitations	—	—	0.238 (0.008)	<0.001	—	—
IADL limitations	—	—	0.156 (0.009)	<0.001	—	—

VI, visual impairment; SE, standard error; CES-D, Center for Epidemiologic Studies Depression scale; ADL, activities of daily living; IADL, instrumental activities of daily living; IPCW, inverse probability of censoring weighting. All models include random intercepts per participant and adjust for age, sex, race/ethnicity, education, log household wealth, hearing impairment, diabetes, current smoking, and time-varying comorbidity burden. The main model additionally adjusts for baseline CES-D score. Sensitivity Analysis 1 additionally adjusts for time-varying ADL and IADL limitations. Sensitivity Analysis 2 applies IPCW to account for informative dropout, with weights truncated at the 99th percentile. All continuous predictors were standardized (*z*-scores). —, not applicable.

### Social vulnerability as an effect modifier

**Figure 3:**
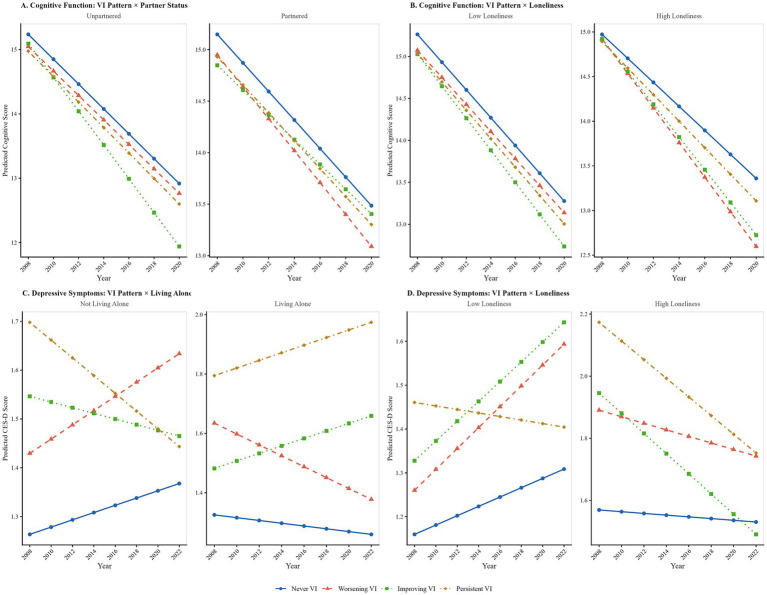
Adjusted predicted trajectories by visual impairment pattern and social vulnerability modifier. Predicted values were derived from linear mixed-effects models including three-way interactions of VI pattern × time × social modifier, adjusted for baseline outcome score, age, sex, race/ethnicity, education, log household wealth, hearing impairment, diabetes, current smoking, and time-varying comorbidity burden. Predictions were generated with continuous covariates set to their sample means (*z* = 0 *after standardiz*ation) and categorical covariates set to their reference categories, with the social modifier varied across the levels shown. **(A)** Shows cognitive trajectories stratified by partner status (unpartnered vs. partnered). **(B)** Shows cognitive trajectories stratified by loneliness level (low vs. high loneliness; high loneliness defined as mean UCLA Loneliness Scale score ≥2). **(C)** Shows depressive symptom trajectories stratified by living arrangement (not living alone vs. living alone). **(D)** Shows depressive symptom trajectories stratified by loneliness level. Loneliness analyses were conducted in the subsample with available loneliness data (*n* ≈ 12,588). VI, visual impairment; CES-D, Center for Epidemiologic Studies Depression scale.

After Benjamini-Hochberg false discovery rate correction, one three-way interaction remained significant for cognitive trajectories and four remained significant for depressive symptom trajectories (Supplementary Tables 1, 2; Figure 3).

For cognitive trajectories, partner status significantly modified the association between Improving VI and cognitive decline (VI × Time × Partner: *β* = 0.176, 95% CI: 0.078 to 0.274, *p* < 0.001, *q* = 0.004). Model-based simple slopes showed that among participants with Improving VI, cognitive decline was steeper in unpartnered individuals (*β* = −0.525 per wave, 95% CI: −0.598 to −0.453, *p* < 0.001) than in partnered individuals (*β* = −0.240 per wave, 95% CI: −0.302 to −0.178, *p* < 0.001), indicating attenuation of the adverse cognitive trajectory in the presence of a partner (Supplementary Table 4). The nominal interaction between Worsening VI and high loneliness did not remain significant after FDR correction (*q* = 0.068).

For depressive symptom trajectories, partner status significantly modified the Worsening VI trajectory (VI × Time × Partner: *β* = 0.047, 95% CI: 0.008 to 0.086, *p* = 0.020, *q* = 0.044). Model-based simple slopes suggested a relatively less favorable depressive trajectory among partnered than unpartnered participants with Worsening VI, although neither subgroup-specific slope was individually significant (Supplementary Table 4).

Living arrangement significantly modified depressive symptom trajectories in both the Improving VI and Persistent VI groups. For Improving VI, living alone was associated with a more positive depressive symptom slope than not living alone (VI × Time × Living Alone: *β* = 0.061, 95% CI: 0.012 to 0.110, *p* = 0.013, *q* = 0.040). For Persistent VI, living alone was likewise associated with a more positive depressive symptom slope (*β* = 0.086, 95% CI: 0.031 to 0.141, *p* = 0.002, *q* = 0.008). In simple-slope analyses, participants with Persistent VI who were not living alone showed declining depressive symptoms over time (*β* = −0.036 per wave, 95% CI: −0.061 to −0.012, *p* = 0.004), whereas those living alone showed a positive but non-significant slope (*β* = 0.026 per wave, 95% CI: −0.021 to 0.072, *p* = 0.276) (Supplementary Table 4).

High loneliness significantly modified the depressive symptom trajectory in the Improving VI group (VI × Time × Loneliness: *β* = −0.083, 95% CI: −0.130 to −0.036, *p* < 0.001, *q* = 0.004). In this group, high loneliness was also associated with higher baseline depressive burden (VI × Loneliness: *β* = 0.208, 95% CI: 0.019 to 0.397, *p* = 0.029). Model-based simple slopes showed a slightly positive depressive symptom slope among participants with low loneliness (*β* = 0.017 per wave, 95% CI: −0.007 to 0.041, *p* = 0.163) and a negative slope among those with high loneliness (*β* = −0.066 per wave, 95% CI: −0.101 to −0.031, *p* < 0.001), indicating a distinct trajectory pattern characterized by higher baseline burden and a more negative subsequent slope in the high-loneliness subgroup (Supplementary Table 4).

### Sensitivity analyses

After additional adjustment for time-varying ADL and IADL limitations, the Improving VI × Time effect on cognition was attenuated and no longer significant (*β* = −0.037, 95% CI: −0.084 to 0.010, *p* = 0.130). The Persistent VI × Time effect on depressive symptoms strengthened (*β* = −0.051, 95% CI: −0.073 to −0.029, *p* < 0.001) (Tables 3,4).

IPCW-weighted analyses yielded results consistent with the primary models: the Improving VI × Time cognitive effect remained significant (*β* = −0.048, 95% CI: −0.095 to −0.001, *p* = 0.046) and the Persistent VI × Time depression effect was similarly robust (*β* = −0.033, 95% CI: −0.055 to −0.011, *p* = 0.002). Attrition analyses indicated selective dropout: compared with completers, non-completers were older, had lower baseline cognitive scores, higher depressive symptom scores, lower household wealth, and a higher prevalence of VI patterns (Supplementary Table 3).

## Discussion

This longitudinal study of 15,393 older U.S. adults examined whether distinct patterns of visual impairment over time are differentially associated with trajectories of cognitive function and depressive symptoms across up to 14 years of follow-up. Three principal findings emerged. First, a binary classification of baseline VI obscured meaningful heterogeneity: the four-group longitudinal classification revealed trajectory patterns that a static, single-time-point exposure would have missed entirely. Second, the Improving VI group was the only VI pattern associated with significantly faster cognitive decline relative to Never VI. Third, social vulnerability modified the cognitive and affective consequences of VI, although these interaction effects varied across indicators and outcomes.

### Comparison with prior literature

Cross-sectional associations between VI and both cognitive impairment and depression in older adults are well established (2–4, 21, 28). Longitudinal evidence regarding the rate of change, however, has been mixed (2, 28, 29). Studies relying on binary VI classifications have reported either modest associations with cognitive decline or null results; our Model A findings align with the former pattern—binary baseline VI was associated with substantially lower cognitive scores at baseline (*β* = −0.653, *p* < 0.001) but not with a significantly different rate of decline (*β* = −0.015, *p* = 0.421). This discrepancy between baseline and slope effects illustrates a broader point: treating VI as a static, dichotomous exposure captures baseline burden but lacks the sensitivity to reveal its dynamic relationship with health trajectories unfolding over more than a decade.

Prior work examining VI longitudinally has typically distinguished only between transient and persistent patterns (2, 6). Our four-group classification extends this approach by separating the direction of change within the transient category—differentiating those whose vision improved from those whose vision worsened—and demonstrates that directionality carries prognostic significance beyond simple chronicity.

### The improving VI paradox

The finding that Improving VI was associated with the steepest cognitive decline (*β* = −0.051 per wave, *p* = 0.040) warrants careful interpretation, as it runs counter to the intuitive expectation that vision recovery should confer cognitive benefit. In cumulative terms, this estimate corresponds to approximately 0.31 additional points of cognitive decline over 12 years, or about 1.1% of the 27-point cognitive scale, suggesting a modest but detectable long-term difference. Several non-mutually-exclusive mechanisms may explain this pattern.

First, the resolution of self-reported VI between 2006 and 2008 may reflect corrective interventions—such as cataract surgery or updated prescriptions—that restored visual acuity without reversing neurodegenerative processes already initiated during the period of impairment (8, 28). A growing body of evidence suggests that even transient periods of reduced sensory input can trigger lasting changes in neural connectivity and cognitive reserve (30, 31). Second, self-reported visual improvement may partly reflect declining metacognitive awareness; individuals in early stages of cognitive decline may become less accurate in appraising their own sensory function (32), leading to an apparent improvement that is artifactual rather than physiological (32). Under this account, what appears as VI “improvement” is itself a cognitive marker. Third, the period of VI in 2006 may have initiated a cascade of social withdrawal and reduced cognitive stimulation whose effects persisted beyond the restoration of vision (7, 33)—a pathway consistent with the “cognitive reserve depletion” hypothesis (30, 34), in which a period of reduced environmental input leaves durable cognitive traces. These explanations should be regarded as hypothesis-generating rather than as mechanisms directly demonstrated by the present study, because information on objective visual acuity, visual correction procedures, metacognitive accuracy, and longitudinal changes in social engagement was not available.

The sensitivity analysis adjusting for ADL and IADL limitations attenuated the Improving VI × Time interaction to non-significance (*β* = −0.037, *p* = 0.130), suggesting that physical functional decline partly accounts for the observed cognitive trajectory. However, the effect was preserved in inverse-probability-of-censoring-weighted analyses (*β* = −0.048, *p* = 0.046), indicating that selective attrition did not drive the finding (27). The attenuation by functional limitations may reflect mediation rather than confounding: VI-related functional decline may be one pathway through which prior visual impairment exerts its downstream cognitive effects—a possibility that formal mediation analysis in future work could directly test. At the same time, reverse causality remains plausible: early cognitive decline may alter the perception or reporting of vision, making apparent VI improvement partly a product of changing self-appraisal rather than true sensory recovery. Taken together, these findings suggest that cognitive monitoring should continue even after successful visual correction, as the neurological consequences of prior impairment may extend well beyond the restoration of acuity (8, 28).

In contrast, Persistent VI—despite carrying the highest baseline disease burden—was not associated with significantly steeper cognitive decline (*β* = −0.013, *p* = 0.630). This null finding should be interpreted cautiously given the relatively modest size of this subgroup (*n* = 868), which may have limited statistical power to detect small but clinically meaningful effects.

### Persistent VI and depressive symptoms

The Persistent VI group had markedly elevated depressive symptoms at baseline (*β* = 0.449, *p* < 0.001) but showed a significant flattening of the depressive trajectory relative to the Never VI group over follow-up (*β* = −0.035 per wave, *p* = 0.002). Over 14 years, this estimate corresponds to an approximately 0.25-point relative reduction on the 8-point CES-D scale, or about 3.1% of the total scale range. This pattern likely reflects a combination of regression toward the mean (25) and genuine psychological adaptation (35). Older adults living with chronically impaired vision may develop compensatory coping strategies over time, and the initial assessment may have captured a period of acute adjustment to disability that subsequently resolved (35). The Worsening and Improving VI groups also exhibited elevated baseline depressive symptoms (*β* = 0.206 and 0.251, respectively), but neither showed a significantly different trajectory over time, suggesting that the adaptive trajectory pattern is most pronounced among those with sustained impairment. Selective survival or attrition may also have contributed to this pattern: participants with persistent VI and more severe depressive burden may have been more likely to die or drop out over follow-up, thereby making the observed trajectory among remaining participants appear more favorable. Although IPCW analyses yielded similar results, residual bias from unmeasured predictors of dropout cannot be excluded.

Notably, the Persistent VI × Time effect on depressive symptoms strengthened after adjustment for ADL and IADL limitations (*β* = −0.051 vs. −0.035 in the primary model, *p* < 0.001). This pattern is inconsistent with mediation by functional limitations and instead suggests that ADL/IADL limitations and depressive symptoms share variance that, when partialed out, reveals a stronger underlying association between persistent VI and depressive trajectory. In other words, the psychological adaptation process among those with persistent VI is partially obscured by co-occurring functional decline in unadjusted models—a statistical pattern that warrants careful interpretation in future work examining the interplay of sensory, functional, and affective health.

### Social vulnerability as an effect modifier

The three-way interaction analyses revealed that social context meaningfully modifies the health consequences of VI. It is worth emphasizing that the main effect of Improving VI on depressive symptom trajectories was not statistically significant (*β* = −0.013, *p* = 0.219), yet significant three-way interactions with loneliness emerged for this same group. This is not contradictory: a non-significant main effect reflects the average trajectory pooled across all social contexts, whereas a significant interaction indicates that the effect is concentrated—and clinically meaningful—within a specific subgroup. When a sample is heterogeneous with respect to social vulnerability, averaging across subgroups can obscure effects that are both present and consequential within them.

After false discovery rate correction, partner status remained a significant modifier of cognitive trajectories only in the Improving VI group (three-way interaction *β* = 0.176, *q* = 0.004). Among participants with Improving VI, those without a partner showed substantially steeper cognitive decline than those with a partner, consistent with a buffering role for close social support (36). By contrast, the nominal interaction between Worsening VI and loneliness on cognitive decline did not remain significant after correction for multiple testing and should therefore be interpreted cautiously.

For depressive symptom trajectories, four three-way interactions remained significant after false discovery rate correction. First, among participants with Worsening VI, partner status was associated with a relatively less favorable depressive trajectory in partnered than unpartnered individuals (three-way interaction *β* = 0.047, *q* = 0.044), although the subgroup-specific slopes themselves were not individually significant. Second, living alone modified depressive trajectories in both the Improving VI (*β* = 0.061, *q* = 0.040) and Persistent VI (*β* = 0.086, *q* = 0.008) groups, suggesting that structural social isolation may shape the affective course of VI even when the average group trajectory is flat or improving (37). In the Persistent VI group, participants who were not living alone showed declining depressive symptoms over time, whereas those living alone did not show the same pattern, consistent with attenuation of the apparent adaptive trajectory in the presence of structural isolation. Third, in the Improving VI group, high loneliness was associated with both higher baseline depressive burden and a more negative subsequent slope (three-way interaction *β* = −0.083, *q* = 0.004). This pattern suggests a distinct trajectory characterized by persistently greater affective burden despite partial symptom improvement over follow-up, rather than a simple amplification of depressive worsening. These findings align with the cognitive reserve (30, 31, 38) and stress-buffering frameworks (13): sustained social engagement may preserve the cognitive stimulation and emotional regulation needed to buffer against the downstream consequences of sensory loss. At the same time, these interaction analyses should be interpreted as exploratory with respect to specific modifier-outcome combinations, particularly where subgroup-specific slopes were imprecisely estimated.

### Strengths and limitations

Strengths of this study include the large, nationally representative sample; the longitudinal exposure classification that captures both chronicity and directionality of VI change; extended follow-up spanning up to seven cognitive assessments and eight depression assessments; and the systematic examination of social vulnerability as an effect modifier. The use of linear mixed-effects models accommodated unbalanced data under the missing-at-random assumption (23, 24), and sensitivity analyses using inverse probability of censoring weighting (26, 27) and ADL/IADL adjustment supported the robustness of the primary findings.

Several limitations should be acknowledged. First, VI was based on self-report rather than objective clinical measurement, which may introduce misclassification. Although self-reported eyesight in the HRS has been validated against health outcomes, misclassification may still be nondifferential, tending to attenuate associations, or differential if mood or cognitive status influenced the perception or reporting of vision. This concern is especially relevant to the Improving VI group, in which apparent improvement may partly reflect reporting variability or changing self-appraisal rather than true physiologic recovery. Second, the two-wave exposure window (2006–2008) captures VI status at only two time points; transitions occurring between or after these assessments were not captured, and the four-group classification may therefore miss more granular temporal dynamics. Because this window was selected primarily for data availability, we were also unable to determine whether VI present in 2006 reflected recent onset or longstanding impairment, or whether additional changes in vision occurred after 2008. Third, despite adjustment for a broad set of covariates, residual confounding by unmeasured factors such as access to eye care, specific ocular diagnoses, or health literacy cannot be excluded, and reverse causality remains possible in that declining cognitive function may itself affect the accuracy of self-reported vision. Fourth, the cognitive composite score, while well validated, primarily captures memory and executive function and may not fully reflect domain-specific effects of VI on processing speed, visuospatial ability, or language. Fifth, random slopes for time were not retained because preliminary models yielded singular fits; as a result, standard errors for time-varying effects may be slightly underestimated. Sixth, loneliness data were available for 81.8% of the analytic sample because of the split-sample Leave-Behind Questionnaire design, and the HRS sample primarily comprises community-dwelling older adults in the United States; therefore, findings from loneliness interaction models and the generalizability of the results should be interpreted accordingly.

## Data Availability

The datasets presented in this study can be found in online repositories. The names of the repository/repositories and accession number(s) can be found at: https://hrs.isr.umich.edu/.
