# Supplementary material for: Longitudinal patterns of visual impairment and trajectories of cognitive function and depressive symptoms in older adults: the moderating role of social vulnerability
Source: Front Public Health. 2026 Jul 29;14:1899072. doi: 10.3389/fpubh.2026.1899072 (PMC13461559; doi:10.3389/fpubh.2026.1899072)
Supplement: Supplementary file 1 [file Table_1.docx]

**Supplementary Table 1. Social Vulnerability as an Effect Modifier of Visual Impairment Patterns on Cognitive Function Trajectories**

| **VI Pattern × Modifier** | **Partnered β (SE)** | **p-value** | **q-value** | **Living Alone β (SE)** | **p-value** | **q-value** | **High Loneliness β (SE)** | **p-value** | **q-value** |
| --- | --- | --- | --- | --- | --- | --- | --- | --- | --- |
| VI pattern × Time (ref: Never VI × Time) |  |  |  |  |  |  |  |  |  |
| Worsening VI × Time | 0.006 (0.037) | 0.867 | — | −0.039 (0.026) | 0.135 | — | 0.009 (0.030) | 0.778 | — |
| Improving VI × Time | −0.139 (0.038) | <0.001 | — | −0.020 (0.028) | 0.487 | — | −0.051 (0.032) | 0.116 | — |
| Persistent VI × Time | −0.009 (0.042) | 0.822 | — | −0.005 (0.030) | 0.881 | — | −0.008 (0.038) | 0.842 | — |
| Social modifier main effect on trajectory (Time × Modifier) |  |  |  |  |  |  |  |  |  |
| Time × Modifier | 0.109 (0.013) | <0.001 | — | −0.123 (0.014) | <0.001 | — | 0.062 (0.014) | <0.001 | — |
| Three-way interactions (VI Pattern × Time × Modifier) |  |  |  |  |  |  |  |  |  |
| Worsening VI × Time × Modifier | −0.039 (0.047) | 0.410 | 0.614 | 0.069 (0.053) | 0.191 | 0.430 | −0.127 (0.052) | 0.015 | 0.068 |
| Improving VI × Time × Modifier | 0.176 (0.050) | <0.001 | 0.004 | −0.112 (0.057) | 0.052 | 0.156 | −0.046 (0.056) | 0.408 | 0.614 |
| Persistent VI × Time × Modifier | 0.015 (0.054) | 0.778 | 0.778 | −0.021 (0.064) | 0.738 | 0.778 | −0.021 (0.059) | 0.722 | 0.778 |

Note. VI = visual impairment; SE = standard error; q-value = Benjamini-Hochberg FDR-adjusted p-value. Each column represents a separate linear mixed-effects model including the three-way interaction of VI pattern × time × social modifier, adjusting for baseline cognitive score, age, sex, race/ethnicity, education, log household wealth, hearing impairment, diabetes, current smoking, and time-varying comorbidity burden. The VI pattern × Time rows show the simple slope for each VI group among the reference category of the modifier (i.e., unpartnered, not living alone, or low loneliness). Three-way interaction terms test whether the modifier significantly changes the VI-trajectory association. High loneliness models used a subsample with available loneliness data (n = 12,588, 81.8%). Bold q-values indicate FDR-significant interactions (q < 0.05).

**Supplementary Table 2. Social Vulnerability as an Effect Modifier of Visual Impairment Patterns on Depressive Symptom Trajectories**

| **VI Pattern × Modifier** | **Partnered β (SE)** | **p-value** | **q-value** | **Living Alone β (SE)** | **p-value** | **q-value** | **High Loneliness β (SE)** | **p-value** | **q-value** |
| --- | --- | --- | --- | --- | --- | --- | --- | --- | --- |
| VI pattern (ref: Never VI) — Baseline level |  |  |  |  |  |  |  |  |  |
| Worsening VI | 0.239 (0.061) | <0.001 | — | 0.166 (0.046) | <0.001 | — | 0.101 (0.053) | 0.059 | — |
| Improving VI | 0.225 (0.063) | <0.001 | — | 0.283 (0.050) | <0.001 | — | 0.168 (0.057) | 0.003 | — |
| Persistent VI | 0.528 (0.064) | <0.001 | — | 0.435 (0.052) | <0.001 | — | 0.301 (0.063) | <0.001 | — |
| VI pattern × Modifier interaction (baseline) |  |  |  |  |  |  |  |  |  |
| Worsening VI × Modifier | −0.065 (0.079) | 0.416 | — | 0.143 (0.088) | 0.106 | — | 0.221 (0.089) | 0.014 | — |
| Improving VI × Modifier | 0.032 (0.085) | 0.703 | — | −0.126 (0.093) | 0.175 | — | 0.208 (0.096) | 0.029 | — |
| Persistent VI × Modifier | −0.168 (0.087) | 0.053 | — | 0.034 (0.097) | 0.723 | — | 0.303 (0.096) | 0.002 | — |
| VI pattern × Time (ref: Never VI × Time) |  |  |  |  |  |  |  |  |  |
| Worsening VI × Time | −0.024 (0.016) | 0.134 | — | 0.014 (0.011) | 0.198 | — | 0.026 (0.013) | 0.039 | — |
| Improving VI × Time | −0.007 (0.016) | 0.649 | — | −0.027 (0.012) | 0.027 | — | 0.024 (0.014) | 0.083 | — |
| Persistent VI × Time | −0.026 (0.018) | 0.147 | — | −0.051 (0.013) | <0.001 | — | −0.029 (0.016) | 0.067 | — |
| Social modifier main effect on trajectory (Time × Modifier) |  |  |  |  |  |  |  |  |  |
| Time × Modifier | 0.030 (0.005) | <0.001 | — | −0.024 (0.006) | <0.001 | — | −0.027 (0.006) | <0.001 | — |
| Three-way interactions (VI Pattern × Time × Modifier) |  |  |  |  |  |  |  |  |  |
| Worsening VI × Time × Modifier | 0.047 (0.020) | 0.020 | 0.044 | −0.042 (0.023) | 0.067 | 0.101 | −0.042 (0.022) | 0.059 | 0.101 |
| Improving VI × Time × Modifier | −0.003 (0.021) | 0.896 | 0.896 | 0.061 (0.025) | 0.013 | 0.040 | −0.083 (0.024) | <0.001 | 0.004 |
| Persistent VI × Time × Modifier | −0.006 (0.023) | 0.778 | 0.875 | 0.086 (0.027) | 0.002 | 0.008 | −0.025 (0.025) | 0.312 | 0.401 |

Note. VI = visual impairment; SE = standard error; CES-D = Center for Epidemiologic Studies Depression scale; q-value = Benjamini-Hochberg FDR-adjusted p-value. Each column represents a separate linear mixed-effects model including the three-way interaction of VI pattern × time × social modifier, adjusting for baseline CES-D score, age, sex, race/ethnicity, education, log household wealth, hearing impairment, diabetes, current smoking, and time-varying comorbidity burden. The VI pattern × Modifier interaction (baseline) tests whether the social modifier changes the baseline difference between VI groups and Never VI. The VI pattern × Time rows show the simple slope for each VI group among the reference category of the modifier (unpartnered, not living alone, or low loneliness). Three-way interaction terms test whether the modifier significantly changes the VI-trajectory association. q-values were calculated separately for depressive symptom outcomes using the Benjamini-Hochberg procedure. High loneliness models used a subsample with available loneliness data (n = 12,588, 81.8%). Partnered = having a spouse or partner; Living alone = single-person household. Bold q-values indicate FDR-significant interactions (q < 0.05).

**Supplementary Table 3. Baseline Characteristics of Completers Versus Non-completers**

| **Characteristic** | **Completers (n=6,528)** | **Non-completers (n=8,865)** | **p-value** |
| --- | --- | --- | --- |
| Age, years, mean (SD) | 64.41 (7.54) | 72.86 (10.10) | <0.001 |
| Baseline cognitive score, mean (SD) | 16.63 (3.93) | 13.90 (4.67) | <0.001 |
| Baseline CES-D score, mean (SD) | 1.17 (1.82) | 1.63 (2.05) | <0.001 |
| Log household wealth, mean (SD) | 11.14 (4.99) | 10.57 (4.91) | <0.001 |
| Loneliness score, mean (SD) | 1.45 (0.53) | 1.50 (0.55) | <0.001 |
| Female, n (%) | 4,084 (62.6) | 5,159 (58.2) | <0.001 |
| Hearing impairment, n (%) | 1,029 (15.8) | 2,184 (24.6) | <0.001 |
| Diabetes, n (%) | 1,038 (15.9) | 2,122 (23.9) | <0.001 |
| Current smoking, n (%) | 745 (11.4) | 1,244 (14.0) | <0.001 |
| Partnered, n (%) | 4,690 (71.8) | 5,081 (57.3) | <0.001 |
| Living alone, n (%) | 1,212 (18.6) | 2,630 (29.7) | <0.001 |
| High loneliness, n (%) | 1,593 (24.4) | 2,518 (28.4) | <0.001 |
| VI pattern, n (%) |  |  | <0.001 |
| Never VI | 5,674 (86.9) | 6,928 (78.1) |  |
| Worsening VI | 340 (5.2) | 702 (8.0) |  |
| Improving VI | 294 (4.5) | 587 (6.7) |  |
| Persistent VI | 220 (3.4) | 648 (7.3) |  |

Note. Completers were defined as participants with available cognitive outcome data at Wave 15 (2020). Non-completers were participants with baseline data at Wave 9 (2008) who were lost to follow-up prior to Wave 15. p-values are from independent-samples t-tests for continuous variables and chi-square tests for categorical variables. Hearing impairment was defined as self-reported hearing rated poor or fair (≤2 on a 5-point scale from 1=poor to 5=excellent). CES-D = Center for Epidemiologic Studies Depression scale; VI = visual impairment.

**Supplementary Table 4. Simple Slopes of Time for FDR-Significant Three-Way Interactions Between Visual Impairment Pattern and Social Vulnerability Modifiers**

| **Outcome** | **Social Modifier** | **Modifier Level** | **VI Pattern** | **Time Slope β (SE)** | **95% CI** | **p-value** |
| --- | --- | --- | --- | --- | --- | --- |
| Cognition | Partnered | Unpartnered | Improving VI | −0.525 (0.037) | [−0.598, −0.453] | <0.001 |
| Cognition | Partnered | Partnered | Improving VI | −0.240 (0.032) | [−0.302, −0.178] | <0.001 |
| Depressive Symptoms | Partnered | Unpartnered | Worsening VI | −0.024 (0.016) | [−0.056, 0.008] | 0.134 |
| Depressive Symptoms | Partnered | Partnered | Worsening VI | 0.023 (0.013) | [−0.003, 0.049] | 0.076 |
| Depressive Symptoms | Living Alone | Not alone | Improving VI | −0.012 (0.012) | [−0.035, 0.011] | 0.319 |
| Depressive Symptoms | Living Alone | Alone | Improving VI | 0.025 (0.021) | [−0.016, 0.066] | 0.231 |
| Depressive Symptoms | Living Alone | Not alone | Persistent VI | −0.036 (0.013) | [−0.061, −0.012] | 0.004 |
| Depressive Symptoms | Living Alone | Alone | Persistent VI | 0.026 (0.024) | [−0.021, 0.072] | 0.276 |
| Depressive Symptoms | High Loneliness | Low | Improving VI | 0.017 (0.012) | [−0.007, 0.041] | 0.163 |
| Depressive Symptoms | High Loneliness | High | Improving VI | −0.066 (0.018) | [−0.101, −0.031] | <0.001 |
| **Outcome** | **Social Modifier** | **Modifier Level** | **VI Pattern** | **Time Slope β (SE)** | **95% CI** | **p-value** |
| Cognition | Partnered | Unpartnered | Improving VI | −0.525 (0.037) | [−0.598, −0.453] | <0.001 |

**Note.** VI = visual impairment; SE = standard error; CI = confidence interval; CES-D = Center for Epidemiologic Studies Depression scale. This table presents simple slopes (time trends per biennial wave) for VI patterns stratified by social modifier levels, limited to three-way interactions that remained statistically significant after Benjamini-Hochberg false discovery rate correction (q < 0.05). VI was defined as self-reported distance eyesight rated poor or fair (eyefr ≤ 2 on a 5-point scale, where 1 = poor and 5 = excellent). VI patterns: Never VI (no VI at either wave), Worsening VI (no VI in 2006, VI in 2008), Improving VI (VI in 2006, no VI in 2008), Persistent VI (VI at both waves). Cognitive function was assessed using the 27-point HRS composite score; depressive symptoms were assessed using the 8-item CES-D scale. Models were adjusted for baseline outcome score, age, sex, race/ethnicity, education, log household wealth, hearing impairment, diabetes, current smoking, and time-varying comorbidity burden. Modifier levels: Partnered = having a spouse or partner; Alone = living in a single-person household; High loneliness = UCLA Loneliness Scale mean score ≥ 2 (range 1–3).
